# Utility of ^123^I-MIBG Standardized Uptake Value in Patients with Refractory Pheochromocytoma and Paraganglioma

**DOI:** 10.22038/AOJNMB.2019.35953.1245

**Published:** 2019

**Authors:** Hiroshi Wakabayashi, Tahahiro Konishi, Hiroto Yoneyama, Anri Inaki, Tomo Hiromasa, Takafumi Yamase, Norihito Akatani, Satoru Watanabe, Hiroshi Mori, Daiki Kayano, Seigo Kinuya

**Affiliations:** 1 Department of Nuclear Medicine, Kanazawa University Hospital, Kanazawa, Japan; 2 Department of Radiological Technology, Kanazawa University Hospital, Kanazawa, Japan

**Keywords:** ^123^I-MIBG, Refractory pheochromocytoma, Paraganglioma

## Abstract

**Objective(s):**

Single-photon emission computed tomography (SPECT) using metaiodobenzylguanidine (MIBG) is an important diagnostic tool for the treatment of refractory pheochromocytoma and paraganglioma (PPGL). Owing to the difficulty of SPECT quantification, the tumour-to-background ratio (TBR) is used to assess disease activity. However, the utility of TBR is limited owing to the background setting. A quantification technique of SPECT/computed tomography (CT) would facilitate image interpretation. This study aimed to assess the relationship between ^123^I-MIBG maximum standardized uptake value (SUV_max_) and TBR and levels of urinary catecholamines and metabolites in patients with refractory PPGL.

**Methods:**

This study included 15 patients with refractory PPGL who underwent ^131^I-MIBG therapy. Overall, 27 ^123^I-MIBG SPECT/CT images were acquired before and after the therapy. Lesions observed on whole-body images were analysed; the maximum number of lesions per scan was 10. ^123^I-MIBG SUV_max_ was semi-automatically calculated using Q. Metrix package (GE Healthcare). TBR was manually calculated according to the following formula: (max count in lesion − max count in background)/max count in background. Background was set in the contralateral area. When a background region of interest could not be set in the area, it was set in the thigh area. Urine was sampled for 24 h to measure catecholamine and metabolite levels. Increases of ≥3-fold were considered abnormal. TBR, ^123^I-MIBG SUV_max_ and urinary catecholamine and metabolite levels were compared using linear regression analysis.

**Results:**

All patients had MIBG-avid lesions, as seen on ^123^I-MIBG SPECT/CT. A significant relationship between ^123^I-MIBG SUV_max_ and TBR was observed (correlation coefficient [r] =0.84, P < 0.0001). In 27 SPECT/CT examinations, normetanephrine (NMN) level was abnormally increased in 51% (14/27), but other catecholamine and other metabolites were abnormally increased in < 26% (7/27). ^123^I-MIBG SUV_max_ strongly correlated with NMN (r=0.76, P < 0.01) and log NMN (r=0.74, P < 0.01).

**Conclusion:**

^123^I-MIBG SUV_max_ demonstrated similar trends as TBR and reflected urinary NMN in patients with refractory PPGL. Semi-automatic quantification of SPECT/CT could be a useful tool for the evaluation of disease activity.

## Introduction

Conventional ^123^I-metaiodobenzylguanidine (MIBG) single-photon emission computed tomography (SPECT) is a well-established nuclear imaging modality used for the staging of refractory pheochromocytoma and paraganglioma (PPGL) (1, 2). In Japan, ^123^I-MIBG has been used as an important diagnostic tool because of low availability of positron emission tomography (PET) tracers and for preparation of ^131^I-MIBG therapy.

^123^I-MIBG scintigraphy can detect catecholamine-secreting lesions associated with refractory PPGL. MIBG, a guanethidine analogue that resembles norepinephrine, can enter chromaffin cells through active uptake or passive diffusion; active uptake occurs via norepinephrine transporters. MIBG that diffuses across the chromaffin cell membrane is then stored in neurosecretory granules containing catecholamine (1, 3, 4). Monitoring the activity of catecholamine-secreting lesions using ^123^I-MIBG scintigraphy has provided important information; excessive catecholamine secretion from tumours could exacerbate patients’ quality of life and prognoses. If the relationship between standardized uptake value (SUV) measurement on ^123^I-MIBG scintigraphy and catecholamine-secreting lesions is explained, an accurate assessment of ^123^I-MIBG uptake would be possible.

To objectively evaluate ^123^I-MIBG accumulation in the clinic, a quantitative index for ^123^I-MIBG scintigraphy is urgently needed. The most common approach for interpreting ^123^I-MIBG scintigraphy at present is visual inspection; therefore, diagnostic performance mainly relies on the experience of physicians. Few studies have focused on quantitative lesion analyses using SUV in the context of ^201^Tl and ^99m^Tc-based scintigraphy performed with an advanced SPECT scanner in combination with computed tomography (CT) (5-7). SUV is calculated as the ratio of the amount of injected dose at the time of injection divided by body weight. The semi-quantitative SUV is increasingly favoured by clinicians seeking to characterise lesions rather than perform visual analysis and calculate the tumour-to-background uptake ratio (TBR).

Therefore, the aim of this study was to assess the relationship between ^123^I-MIBG maximum SUV (SUV_max_) and semi-quantitative index of TBR and levels of catecholamines and metabolites in patients with refractory PPGL.

## Methods

***Patients***

This retrospective study included 15 patients [female, n=5; male, n=10; median age, 52.6±11.8 (range, 24 to 73) years] with refractory pheochromocytoma (n=7) and paraganglioma (n=8) who underwent ^131^I-MIBG therapy. In total, 27 ^123^I-MIBG SPECT/CT scans were acquired before and after the therapy. ^123^I-MIBG SPECT/CT was performed in 1 of 7 patients, 2 of 4 patients and 3 of 4 patients. The lesions observed in whole-body images were analysed; the maximum number of lesions per scan was 10 with a maximum of 5 per organ. This analysis was performed in compliance with the Declaration of Helsinki and approved by the ethics commission of Kanazawa University Hospital.

^123^ ***I-MIBG SPECT/CT***

After patients underwent whole-body planar imaging, SPECT images were obtained. SPECT imaging was used to visualise the thoracic, abdominal and pelvic areas in whole body scan images 24 h after ^123^I-MIBG injection (FUJIFILM RI Pharma Co., Ltd, Tokyo, Japan, 222 MBq). SPECT data were acquired with a 128×128 matrix and 60 projections (20 sec per view). Following SPECT data acquisition, CT transmission scanning for topography was performed. The scan parameters for CT were 120 kV and a rotation time of 0.5 sec. Tube current was reduced to 40 mA to minimise radiation exposure. SPECT and CT were acquired during shallow breathing. While SPECT and CT scans were being acquired, patients were lying stably in the supine position. The interval between SPECT and CT was within a few minutes. SPECT was reconstructed using a 3-dimensional ordered subsets expectation maximisation algorithm with resolution recovery, scatter and CT-based attenuation correction (AC). The number of subsets and iteration was 10 and 3, respectively. SPECT and CT data were analysed and co-registered using a Xeleris workstation (GE healthcare, Tokyo, Japan).

***Image evaluation***

The targeted lesion met the following criteria: 1) a lesion must be confirmed clearly on ^123^I-IBG whole-body scintigraphy; 2) a lesion must be confirmed clearly on ^123^I-IBG SPECT; 3) a lesion must be observed on CT and 4) a background region must be set on the SPECT image.

^123^I-MIBG SUV_max_ was calculated semi-automatically by Q. Metrix package (GE Healthcare). SPECT sensitivity was calculated using the following equations: 

$\mathrm{SPECT s}\mathrm{ensitivity}=\left(\frac{N}{t_{d}\times A_{0}}\right)\times e^{\left[\frac{\ln(2)\times (T_{s}-T_{0})}{T_{\mathrm{half}}}\right]}$

where N is total number of count, T_d_ is data acquisition time (sec), A_0 _is absolute activity (Bq), Ts is start time of acquisition, T_0 _is time of activity measurement and T_half_ is physical half-life of ^123^I (sec). Pixels uptake in a voxel of interest (VOI) was calculated using the following equations:

SPECT image pixels uptake Bqml=37×103×1SPECT sensitivity60durationml

where, Duration is SPECT acquisition time (sec) and ml is volume of VOI. The SUV can be calculated using the following equations: 

SUVgml=SPECT image pixels uptake Bqml×weight in gramsActual activity

where the Actual activity (Bq) is the dose at injection time, assuming a tissue specific gravity of 1.0. 

TBR for max count was measured for targeted lesions as follows: 

$\mathrm{TBR}=\frac{(\max\mathrm{count in lesion}-\max\mathrm{count in background})}{\max\mathrm{count in background}}$

Background lesions were classified as follows: 1) liver: if a target lesion was in the liver and liver uptake was normal; 2) ipsilateral area: if a lesion was not in the midline and uptake in the ipsilateral area was normal; 3) thigh: if a lesion was not in the thigh and abnormal uptake was not observed and 4) intracranial cavity: if a lesion was not in the intracranial cavity and abnormal uptake was not observed.

***Urinary hormones***

Urinary catecholamines and metabolites were collected to measure disease activity. The levels of urinary catecholamines and associated metabolites were assessed for 24 h by measuring the concentrations of adrenaline (AD), noradrenaline (NA), dopamine (DO), homovanillic acid (HVA), vanillylmandelic acid (VMA), metanephrine (MN) and normetanephrine (NMN). The normal ranges for the concentrations were 1–23 μg/day for AD, 29–120 μg/day for NA, 100–1000 μg/day for DO, 1.6–5.5 mg/day for HVA, 1.4–4.9 mg/day for VMA, 0.05–0.20 mg/day for MN and 0.10–0.28 mg/day for NMN. Increases of ≥3-fold above upper cut-offs (false-positives) are rare and defined as abnormal increases (8). We also used the logarithms of the variables because the values of the levels of urinary catecholamines and associated metabolites covered a large range of scales.

**Figure 1:**
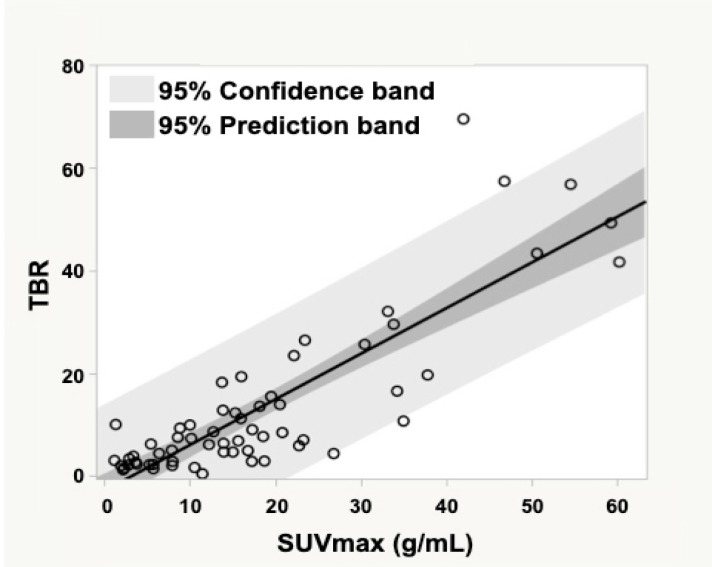
Correlation between SUV_max_ and TBR

SUV_max_ correlated with TBR (Y=0.88x−2.8, correlation coefficient=0.84, P <0.0001). SUV_max_: maximum standardized uptake value and TBR: Tumour-to-background ratio

**Figure 2:**
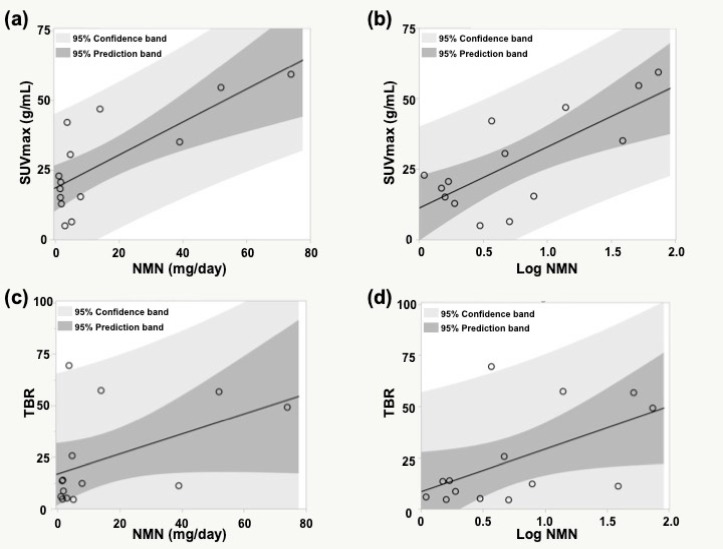
Comparisons between ^123^I-MIBG SUV_max_, TBR and NMN

^123^I-MIBG SUV_max_ correlated with NMN (Y=0.5x+18.5, correlation coefficient [r]=0.76, P < 0.01) (a) and log NMN (Y=21.6x+11.0, r=0.74, P < 0.01) (b). TBR correlated with NMN (Y=0.4x+16.8, r=0.46, P=0.08) (c) and log NMN (Y=20.7x+8.4, r=0.53, P < 0.05) (d). MIBG: metaiodobenzylguanidine, TBR: Tumour-to-background ratio, NMN normetanephrine, and SUV_max_: maximum standardized uptake value

***Reproducibility of TBR reproducibility***

We assessed the reproducibility of TBR measurements. Two physicians with experience in nuclear medicine analysed all studies separately. Manual steps such as drawing region of interests (ROIs) over the tumour uptake and background may have resulted in inter-operator variability.

***Statistical analysis***

For statistical analysis, we used a statistical software package (JMP® SAS Institute Inc., Cary, NC, USA). Correlations among TBR, urinary levels of catecholamines and metabolites and ^123^I-MIBG SUV_max_ were compared using linear regression analysis. Intra-observer reproducibility of TBR was assessed using linear regression analysis. A P value of <0.05 was considered statistically significant.

## Results

All patients had MIBG-avid lesions, as seen on ^123^I-MIBG SPECT/CT. For 60 targeted MIBG-avid lesions, there were 15/60 (25%) in primary and relapse tumours, 18/60 (30%) in bone, 17/60 (27.5%) in lymph node, 2/60 (5%) in liver and 8/60 (20%) in lung metastases.

^123^I-MIBG SUV_max_ (mean, 17.9±14.6 g/mL; range, 1.9–60.2 g/mL) and TBR (mean, 13.1±15.4; range, 0.4–69.4) were separately calculated. A strong correlation was observed between ^123^I-MIBG SUV_max_ and TBR (Figure1).

***Reproducibility of TBR***

We set background ROI, except ipsilateral area, on the thigh for 21 lesions and on liver for 2 lesions. High inter-observer reproducibility was confirmed (correlation coefficient=0.89, P < 0.0001).

***Urinary hormones***

Mean urinary catecholamine hormones were observed in all patients. They were as follows: AD (mean, 40.6 ± 133.6 μg/day; range, 1.3–679 μg/day), NA (mean, 850±2,295 μg/day; range, 73.4–12,100 μg/day), DO (mean, 1,872 ± 1,649 μg/day; range, 550–7900 μg/day), HVA (mean, 22.7 ± 47.6 mg/day; range, 2.7–214 mg/day), VMA (mean, 5.3 ± 3.3 mg/day; range, 2.6–18 mg/day), MN (mean, 0.6 ± 1.7 mg/day; range, 0.03–8.0 mg/day) and NMN (mean, 7.9 ± 17.9 mg/day; range, 0.14–74 mg/day).

In 27 SPECT/CT examinations, an abnormal increase (>3 times of upper limit of normal) was confirmed in 2/27 (3.7%) in AD, 7/27 in (25.9%) in NA, 5/27 in (18.5%) in DO, 1/27 in (3.7%) in HVA, 7/27 in (25.9%) in VMA, 4/27 in (14.8%) in MN and 14/27 in (51.8%) in NMN.

We selected NMN to analyse the correlation with the highest ^123^I-MIBG SUV_max_ (mean, 24.7 ± 17.8 g/mL; range, 3.8–60.2 g/mL) and TBR (mean, 18.5 ± 19.9; range, 1.3–69.4) in each patient (n = 14). NMN (mean, 15.0 ± 22.9 mg/day; range, 1.1–74 mg/day) significantly correlated with ^123^I-MIBG SUV_max_ but not with TBR (Figure 2). Log NMN (mean, 0.7±0.6; range, 0.04–1.86) was strongly correlated with ^123^I-MIBG SUV_max_ and moderately with TBR.

## Discussion

To the best of our knowledge, this is the first study to evaluate the relationship between SUV_max_ and TBR and NMN in patients with refractory PPGL. The results showed that SUV_max _strongly correlated with TBR and NMN levels. SUV_max_ would represent a novel imaging biomarker that can be used to monitor the activity of catecholamine-secreting tumours.

Previous studies showed no association of the ^123^I-MIBG uptake ratio (9), percentage uptake of ^123^I-MIBG (3) and ^123^I-MIBG uptake grade scale (10) with catecholamine and metabolite levels in patients with primary and refractory PPGL. We analysed patients with abnormally increased NMN levels, whereas previous studies analysed all patients regardless of the NMN levels. Approximately 50% of our patients had an abnormally increased NMN (>3 times of the upper limit of normal). Less secretion of catecholamine owing to dedifferentiation of tumour cells, loss of specific neurotransmitter transporters, episodic secretion of catecholamine from tumours and storage of catecholamine metabolite inside the tumours might not cause an abnormal increase of catecholamine and metabolite (11-13). Thus, our findings may be applied to patients with catecholamine-secreting tumours.

We used the highest SUV_max_ for evaluating the catecholamine-secreting tumours. SUV_max_ represents the highest activity of a single pixel within the VOI and is the most common clinical parameter used to assess tracer accumulation in tumours. Although SUV_max_ does not reflect the whole tumour burden, it is measurable without a complicated procedure to decide the border values for setting VOI in multiple organ metastases. Recently, the relationship between tumour burdens quantified by PET tracer and catecholamine metabolites has been reported. Tumour burdens quantified using ^18^F-fluoro-l-dopa (^18^F-DOPA) (14) or ^68^Ga-NaI-octreotide (^68^Ga-DOTANOC) (15) correlated with catecholamine and metabolite levels in patients with primary and refractory PPGL. These relationships suggest that both SUV_max_ and tumour burden support the understanding of the tumour condition in patients with refractory PPGL.

^123^I-MIBG scintigraphy is still essential in the clinical setting, whereas ^123^I-MIBG scintigraphy was negative in approximately 50% of refractory PPGL for the limited spatial resolution of SPECT (12). Recently, PET tracers have been used to detect non-MIBG-avid lesions in patients with PPGL, reflecting the varied character of PPGL tissue and the superior resolution of PET (2, 16), but ^123^I-MIBG also has been used in hospitals that cannot use the PET tracers. ^123^I-MIBG scintigraphy is necessary for dosimetry before starting the treatment of patients with refractory PPGL. In addition, the information of false-negative ^123^I-MIBG in patients with refractory PPGL could suggest a more aggressive disease course and was frequently linked to the presence of succinate dehydrogenase subunit B (SDHB) gene mutation (17). ^123^I-MIBG scintigraphy remains useful for the assessment and evaluation of refractory PPGL.

The commercial SPECT/CT device provided a simple way to achieve semi-quantification with proper quality control consisting of dose calibration and measurement of the detector sensitivity. For the quality control, Bailey et al. mentioned that a complicated process is required in SPECT systems compared with PET system because system sensitivity, image reconstruction and correction methods vary depending on radionuclides in the SPECT system (18). Even considering the above, the quantified SPECT will be a powerful tool to monitor biological processes, such as evaluation of the catecholamine-secreting tumours in our study.

## Conclusion

^123^I-MIBG SUV_max_ demonstrated similar trends as TBR and reflected urinary NMN. The semi-automatic quantification value presented above, ^123^I-MIBG SUV_max_, is an important tool for the evaluation of catecholamine-secreting tumours in patients with increased levels of NMN that are attributed to refractory PPGL.

## Data Availability

The datasets generated during the current study are included in this published article.
